# Targeting autophagy increases the efficacy of proteasome inhibitor treatment in multiple myeloma by induction of apoptosis and activation of JNK

**DOI:** 10.1186/s12885-022-09775-y

**Published:** 2022-07-06

**Authors:** Azam Salimi, Kema Marlen Schroeder, Mirle Schemionek-Reinders, Margherita Vieri, Saskia Maletzke, Deniz Gezer, Behzad Kharabi Masouleh, Iris Appelmann

**Affiliations:** 1 Department of Hematology, Oncology, Hemostaseology and Stem Cell Transplantation, RWTH Aachen University Hospital, Pauwelsstrasse 30, 52074 Aachen, Germany; 2 Institute of Laboratory Medicine, Universities of Giessen and Marburg Lung Center (UGMLC), Philipps University Marburg, German Center for Lung Research (DZL) Marburg, Marburg, Germany

**Keywords:** Autophagy, Multiple myeloma, Proteasome inhibition, Jnk

## Abstract

**Background:**

The therapeutic armamentarium in multiple myeloma has been significantly broadened by proteasome inhibitors, highly efficient means in controlling of multiple myeloma. Despite the developments of therapeutic regimen in treatment of multiple myeloma, still the complete remission requires a novel therapeutic strategy with significant difference in outcomes. Proteasome inhibitors induce autophagy and ER stress, both pivotal pathways for protein homeostasis. Recent studies showed that the IRE1α-XBP1 axis of the unfolded protein response (UPR) is up-regulated in multiple myeloma patients. In addition, XBP1 is crucial for the maintenance of viability of acute lymphoblastic leukemia (ALL).

**Results:**

We analyzed the efficacy of targeting IRE1α-XBP1 axis and autophagy in combination with proteasome inhibitor, ixazomib in treatment of multiple myeloma. In this present study, we first show that targeting the IRE1α-XBP1 axis with small molecule inhibitors (STF-083010, A106) together with the ixazomib induces cell cycle arrest with an additive cytotoxic effect in multiple myeloma. Further, we examined the efficacy of autophagy inhibitors (bafilomycin A, BAF and chloroquine, CQ) together with ixazomib in multiple myeloma and observed that this combination treatment synergistically reduced cell viability in multiple myeloma cell lines (viable cells Ixa: 51.8 ± 3.3, Ixa + BAF: 18.3 ± 7.2, Ixa + CQ: 38.4 ± 3.7) and patient-derived multiple myeloma cells (Ixa: 59.6 ± 4.4, Ixa + CQ: 7.0 ± 2.1). We observed, however, that this combined strategy leads to activation of stress-induced c-Jun N-terminal kinase (JNK). Cytotoxicity mediated by combined proteasome and autophagy inhibition was reversed by addition of the specific JNK inhibitor JNK-In-8 (viable cells: Ixa + BAF: 11.6 ± 7.0, Ixa + BAF + JNK-In-8: 30.9 ± 6.1).

**Conclusion:**

In this study we showed that combined inhibition of autophagy and the proteasome synergistically induces cell death in multiple myeloma. Hence, we consider the implication of pharmaceutical inhibition of autophagy together with proteasome inhibition and UPR-directed therapy as promising novel in vitro treatment strategy against multiple myeloma.

**Supplementary Information:**

The online version contains supplementary material available at 10.1186/s12885-022-09775-y.

## Introduction

Multiple myeloma (MM) is characterized by monoclonal proliferation of plasma cells mostly within the bones often leading to local destruction. Plasma cells play a crucial role in the mammalian immune response which diminishes in its specificity due to the monoclonality of MM cells, thereby leading to an acquired immunodeficiency [1, 2]. MM accounts for about 10% of all hematologic cancers, with a particularly high incidence in adults above 50 years of age [3]. MM cells depend on proteostasis for their survival because of their increased production and accumulation of non-functional immunoglobulin (Ig) [4, 5]. Proteostasis is a complex network with three major branches: the ubiquitin–proteasome system (UPS), the unfolded protein response (UPR) and autophagy. The UPS is the essential network responsible for degradation of unwanted proteins and is targeted by proteasome inhibitors, which are efficient and widely used for the treatment of MM [5–9].

The UPR mediates its function through three subordinated pathways, namely inositol-requiring enzyme 1 alpha (IRE1α), protein kinase RNA-like ER kinase (PERK) and activating transcription factor 6 (ATF6) [10]. The stress sensors IRE1α, PERK and ATF6 are dissociated from the endoplasmic reticulum (ER) chaperone heat shock 70 kDa protein 5/78 kDa glucose-regulated protein (HSPA5/GRP78) thereby activating the UPR pathway. Activated PERK causes phosphorylation of the eukaryotic translation initiation factor 2α (eIF2α) at serine 51. ATF6 in its active form invokes a variety of downstream genes involved in ER-associated degradation (ERAD). The most conserved UPR pathway is mediated by IRE1α and contains an endoribonuclease (RNAse) and a kinase domain. The RNAse domain reduces the ER stress load by splicing *XBP1* mRNA through removal of a 26-nucleotide intron. IRE1α also governs microRNA biogenesis and degradation of specific mRNA during UPR activation by regulated IRE1-dependent decay (RIDD) [10–13]. Our recently published findings demonstrate that high risk subsets of acute lymphoblastic leukemia are vulnerable to IRE1α based therapy and that genetic and pharmacological inhibition of IRE1α negatively affects the survival of ALL cells [14, 15]. Other studies revealed the importance of the UPR in maintaining malignancy [16], and IRE1α–directed therapy has been successfully applied in triple-negative breast cancer and multiple myeloma [17, 18]. Treatment with the proteasome inhibitors bortezomib and ixazomib influences the originating microenvironment of MM cells (i.e. the bone marrow niche) through an up-regulation of the UPR providing a rationale for an additional UPR targeting in multiple myeloma [19, 20].

Autophagy is strongly activated as a response to proteasome inhibition and represents a strategy within the cell to circumvent drug-induced interruption of proteostasis. As an adaptive pathway it thereby supports the survival of malignant cells [21–23] and can be targeted by specific inhibitors. In our study, we treated MM cell lines KMS11 and RPMI-8226 with ixazomib in combination with UPR inhibitors leading to a substantial decrease of cell survival and proliferation. Addition of autophagy inhibitors as a third substance generated a significant increase of the strong cytotoxic effect. Attacking multiple myeloma cells by inhibition of the proteasome, the UPR and autophagy together is consecutively delineated as a promising in vitro treatment strategy in our study.

## Material and methods

### Human cell culture

Human cell lines KMS11 and RPMI-8226 were originally obtained from JCRB Cell Bank (Japanese Collection of Research Bank) and DSMZ (German Collection of Microorganisms and Cell Cultures), Braunschweig, Germany, respectively. The human mesenchymal stem cell line immortalized by expression of the telomerase reverse transcriptase gene (hMSC-TERT) was kindly provided by Dr. Rebecca Schneider-Kramann (RWTH Aachen). All cell lines were authenticated via STR profiling by Multiplexion (Heidelberg, Germany) [24]. All cell lines were cultured as described previously [15]. The inhibitory drugs comprising STF-083010, A106, bafilomycin A1 and chloroquine were purchased from Sigma Aldrich® and ixazomib (MLN97098) was kindly provided by Takeda® (Cambridge, MA, USA).

### Colony formation assay

Primary bone marrow (BM) samples from patients with MM were provided by RWTH centralized BioMaterial Bank (cBMB). Mononuclear cells (MNCs) from these samples were isolated by gradient centrifugation with Ficoll paque (density 1.077 g/mL). The MNCs were cultured in Iscove´s Modified Dulbecco´s Medium (IMDM) with GlutaMAX® containing 10% fetal bovine serum (FBS, Gibco®), 100 IU/mL penicillin and 100 μg/mL streptomycin (Gibco®) were supplemented with 2 mM l-glutamine, 10^−4^ M 2-mercaptoethanol, 10 ng/mL (rh)IL-6, 10 ng/ml rhSCF, 10 ng/mL rhIL-3, 20 ng/mL G-CSF and 10 ng/mL FLT3-ligand (Immunotools®) in an incubator with a humidified atmosphere of 5% CO2 at 37 °C. For the human CFU assays, 80% methylcellulose without cytokines (Methocult, H4230, Stem Cell Technologies®), 20% IMDM, 10^−4^ M 2-mercaptoethanol, 2 mM l-glutamine were supplemented with 50 ng/ml rhSCF, 10 ng/ml rhIL-3, 10 ng/ml rhGM-CSF, 3 U/ml rhEPO (Immunotools®) and 1% penicillin/streptomycin. 5 $\times$ 10^4^ of pretreated BM MNCs/mL were incubated at 37 °C for 48 h with 5% CO_2_ for 14 days and colonies were counted using inverted light microscopy.

### Flow cytometry

Cell viability was measured using propidium iodide (PI) staining (1 μg/mL of PI, Sigma Aldrich®). Apoptosis was measured using flow cytometric quantification of Annexin-V/PI fraction as described in Supplementary Material. Cell cycle was assayed by flow cytometric quantification of DNA content using PI staining. To synchronize the cell cycle progression at a specific phase of the cell cycle, cells were deprived of FBS in culture medium for 16 h and followed by culture conditions as described above with FBS to re-initiate their cell cycle. Cells were resuspended and permeabilized as described in Supplementary Material.

### SDS-PAGE and western blot analysis

Cells were extracted, loaded and transferred as described in Supplementary Material. As primary antibodies, beta actin, P21 (Abcam®), PARP, p27, phospho-SAPK/JNK, BIM, Caspase-3, cleaved -caspase-3, Beclin-1, LC3A/B (all manufactured by Cell Signaling Technology®) were applied. The proteins were developed with PCA-ECL solution (100 mM Tris–HCL, pH 8.8, 2.5 mM luminol, 0.198 mM p-coumaric acid and 0.2% v/v hydrogen peroxide (Sigma Aldrich®). The protein bands were detected by enhanced chemiluminescence (ECL) detection system (Vilber®).

### Quantitative real-time PCR

RNA isolation and cDNA generation was performed as described previously [15]. Quantitative real-time PCR was performed using the iTaq Universal SYBR Green supermix (Bio-Rad®). Gene expression assays (Applied Biosystems®; ABI7500 FAST real-time PCR) were performed according to manufacturer’s instructions. Endogenous expression of *COX6B* was used for normalization and relative quantification of target gene expression was calculated by the comparative threshold cycle method. Triplicates were measured for each tested condition. Quantitative data are expressed as mean ± SD. Differences between values obtained from each condition were considered statistically significant for values of *p* < 0.05. Primer sequences are listed in Supplementary Table 1.

### Statistical analysis

Statistical analyses were performed using two-tailed student’s *t* test and ANOVA by GraphPad Prism (GraphPad Software, Inc.). The Bliss formula was used to analyze the efficacy of drug combination in comparison with monotherapy, using equation: *Yab*,*P* = (*Ya* + *Yb*)*-* (*Ya* × *Yb*), which defines the concepts of synergy, antagonism and additive effects between two inhibitory drugs [25].

## Results

### Inhibition of IRE1α sensitizes multiple myeloma cells to ixazomib under support of mesenchymal stromal cells (MSCs)

**Fig. 1:**
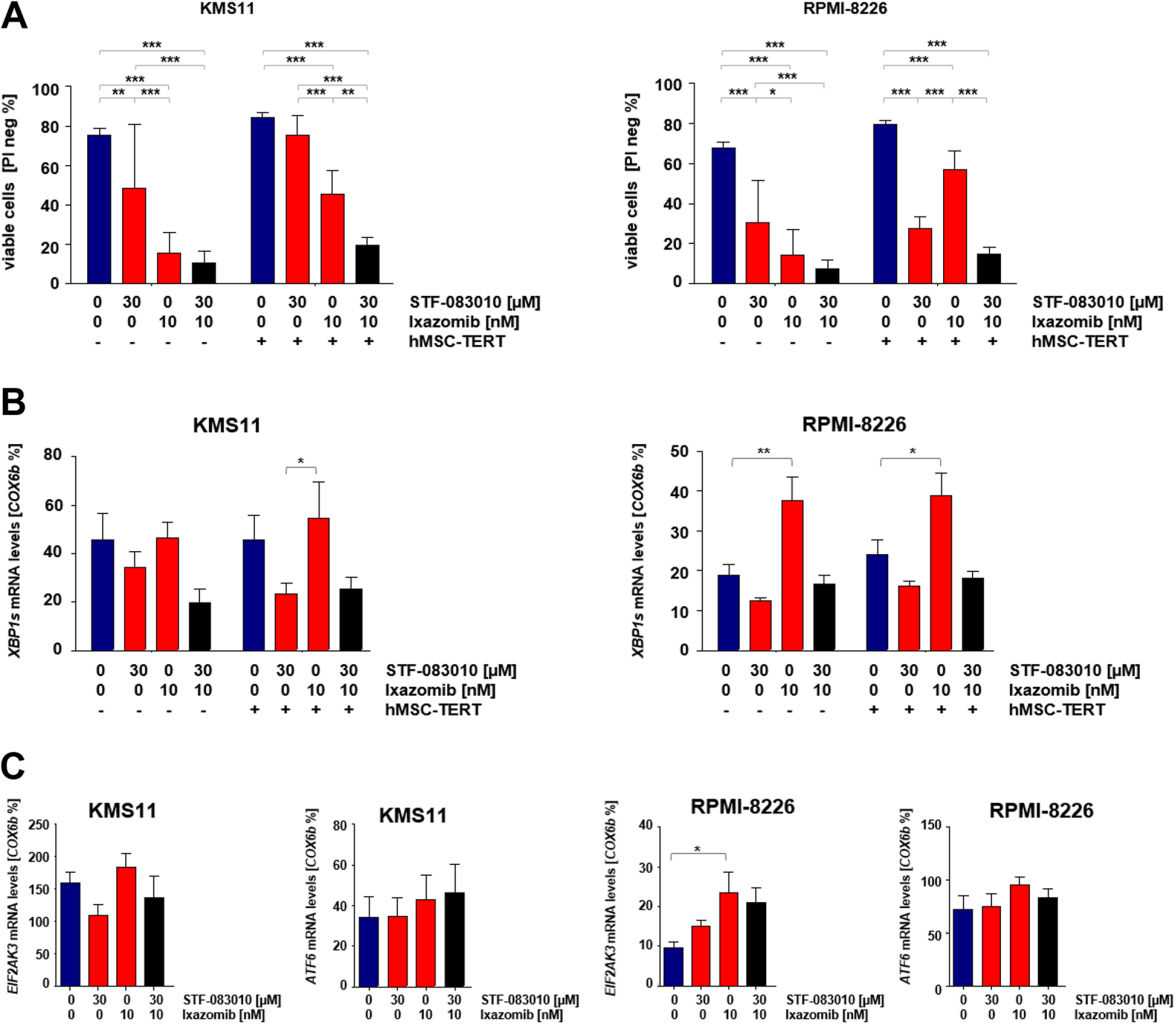
IRE1α inhibitors in combination with ixazomib have synergistic effects on cytotoxicity of multiple myeloma cells under support of mesenchymal stromal cell line (hMSC-TERT).** A** KMS11 and RPMI-8226 cells were treated with 10 nM ixazomib alone or combined with 30 μM STF-083010 for five days with or without support of immortalized stromal cells (hMSC-TERT), (*n* = 3). Cell viability were measured by PI staining. **B** Total RNA was extracted after 16 h of treatment as described above. **B**, **C** *XBP1s*, *ATF6* and *EIF2AK3* mRNA levels were assessed using RT-qPCR, (*n* = 3). *P* values were calculated by two-way analysis of variance (ANOVA) for A, B and one-way analysis for C. A *P* value of less than 0.05 was considered statistically significant for all analyses. (*p < 0.05, **p < 0.01, ***p < 0.001 versus control)

We investigated the capacity of bone marrow MSCs to modulate viability and proliferation of MM cells upon treatment with ixazomib and its combination with selective inhibitors of the IRE1α RNAse domain (STF-083010, A106). We employed hTERT-MSCs with a strong supportive capacity for hematopoesis as a representative model for human bone marrow derived MSCs. MSCs alone did not alter viability of MM cells but treatment with ixazomib supported survival and tumor cell growth compared to culture without MSC support. Interestingly, the combination of ixazomib with STF-083010 and A106, respectively, significantly reduced the viability of multiple myeloma cells even when protected by bone marrow MSCs. This implicates that inhibition of IRE1α in combination with ixazomib abrogates the supportive effect of the bone marrow microenvironment for the survival of MM cells (Fig. 1A, S1A). We furthermore investigated the efficacy of our drug combination in hypoxic conditions to mimic the hypoxic bone marrow niche and measured viability of MM cells with single and dual treatment under normoxic (20% O_2_) versus hypoxic (1% O_2_) conditions. The IRE1α inhibitor STF-083010 did not reduce cell viability in combination with ixazomib under hypoxic conditions (Figure S1B). We then screened the expression signature of major UPR genes, namely *XBP1s*, *EIF2AK3* and *ATF6,* upon treatment with ixazomib as a single agent and in combination with STF-083010. Quantitative RT-PCR analysis showed that ixazomib treated cells only slightly increased mRNA expression of *EIF2AK3* and *ATF6* while the *XBP1s* mRNA level was strongly up-regulated upon treatment with ixazomib (Fig. 1B, C) suggesting that MM cells activate the IRE1α-XBP1 axis as an adaptive pathway shielding against ixazomib-induced cell death.

### IRE1α inhibitors in combination with ixazomib arrest the cell cycle at G_1_ and induce expression of apoptotic effectors

**Fig. 2:**
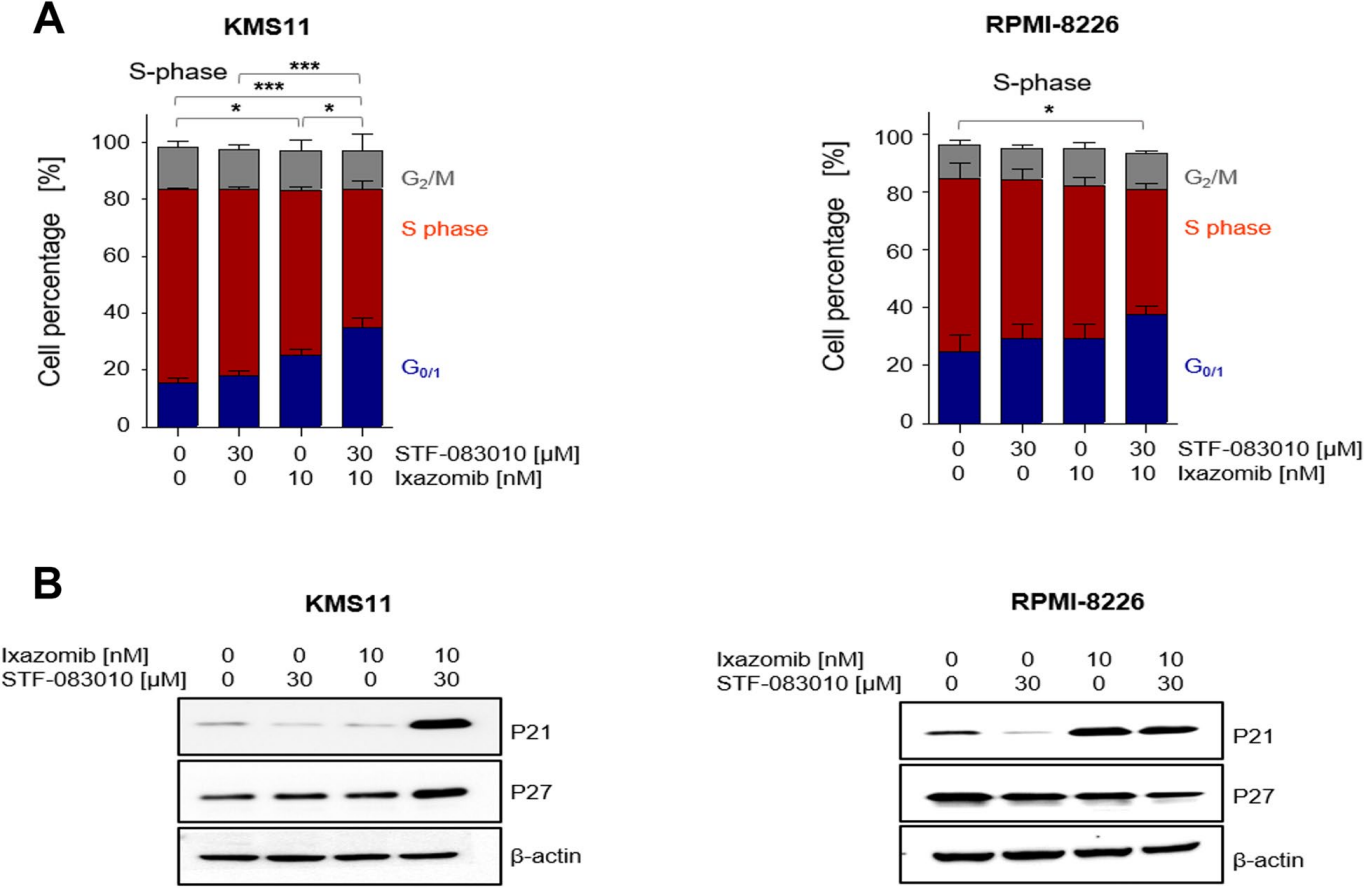
Ixazomib in combination with STF-083010 arrests cell cycle at G_1_ phase. Both KMS11 and RPMI-8226 cells were incubated for 16 h in absence of FBS, followed by the treatment strategy described in Fig. 1 for 16 h), (*n* = 3). **A** Cells were harvested and DNA content was assessed by PI staining. *P* values were calculated by one-way analysis of variance (ANOVA). A *P* value of less than 0.05 was considered statistically significant for all analyses (*p < 0.05, **p < 0.01, ***p < 0.001). **B** Using western blot, protein levels of cell cycle negative regulators p21^CIP1^, p27.^KIP1^ and β-actin as loading control were measured in KMS11 and RPMI-8226 after 16 h of treatment

**Fig. 3:**
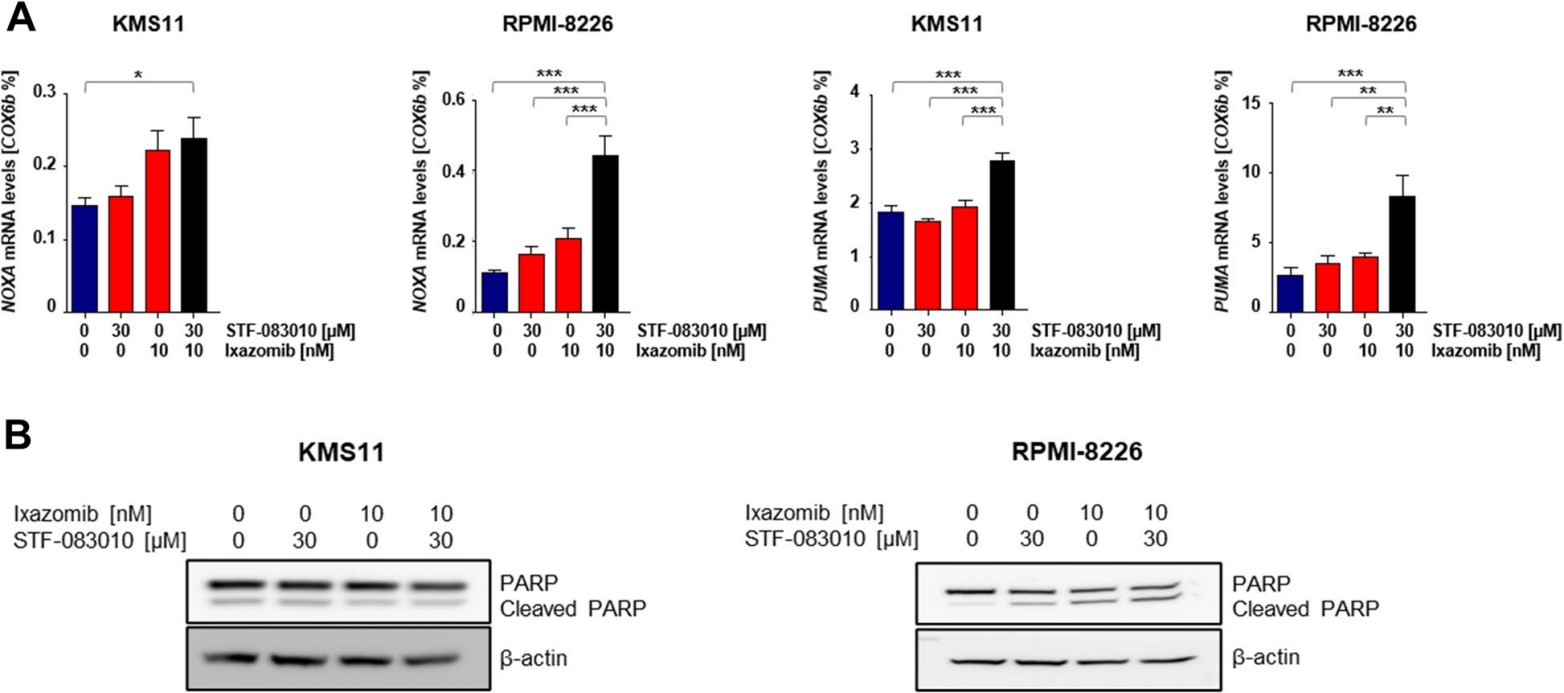
STF-083010 in combination with ixazomib initiates apoptosis in multiple myeloma cell lines. KMS11 and RPMI-8226 cells were treated as described above for 16 h, (*n* = 3). **A** Using RT-qPCR, *PUMA* and *NOXA* mRNA expression levels were measured. *P* values were calculated by one-way analysis of variance (ANOVA). A *P* value of less than 0.05 was considered statistically significant for all of the analysis (*p < 0.05, **p < 0.01, ***p < 0.001). **B** Protein levels of cleaved-PARP, PARP and β-actin as loading control were detected in KMS11 and RPMI-8226 using western blot

The overexpression of the cell cycle regulator cyclin D1 is an important feature of MM cells [4, 26]. We showed that STF-083010 in combination with ixazomib causes accumulation of cells in the G_0/1_ phase of the cell cycle (Fig. 2A). Next, we examined cell cycle regulation of MM cells on the protein level. Combination treatment arrests cell cycle progression at G_1_ and significantly increases expression levels of G_1_ phase regulators, p21^CIP1^ and p27^KIP1^(Fig. 2B, S2A), indicating a reduction of proliferation after addition of IRE1α inhibitors to treatment with ixazomib. We then measured expression levels of the caspase substrate poly (ADP-ribose) polymerase (PARP) and its cleaved form as an indicator of caspase activation. Our combination therapy increased proteolytic cleavage of PARP in RPMI-8226 cells in comparison to single treatment (Fig. 3B, S2B). In addition, pro-apoptotic markers *PUMA* and *NOXA* as ER stress responsive genes [27, 28] were assessed by quantitative RT-PCR. Combination treatment resulted in significantly increased expression of *PUMA* and *NOXA*, suggesting that both are substantially activated in response to our combination regimen to induce cell death in MM cells (Fig. 3A).

### Ixazomib in combination with autophagy inhibitors synergizes cytotoxicity in multiple myeloma cells

**Fig. 4:**
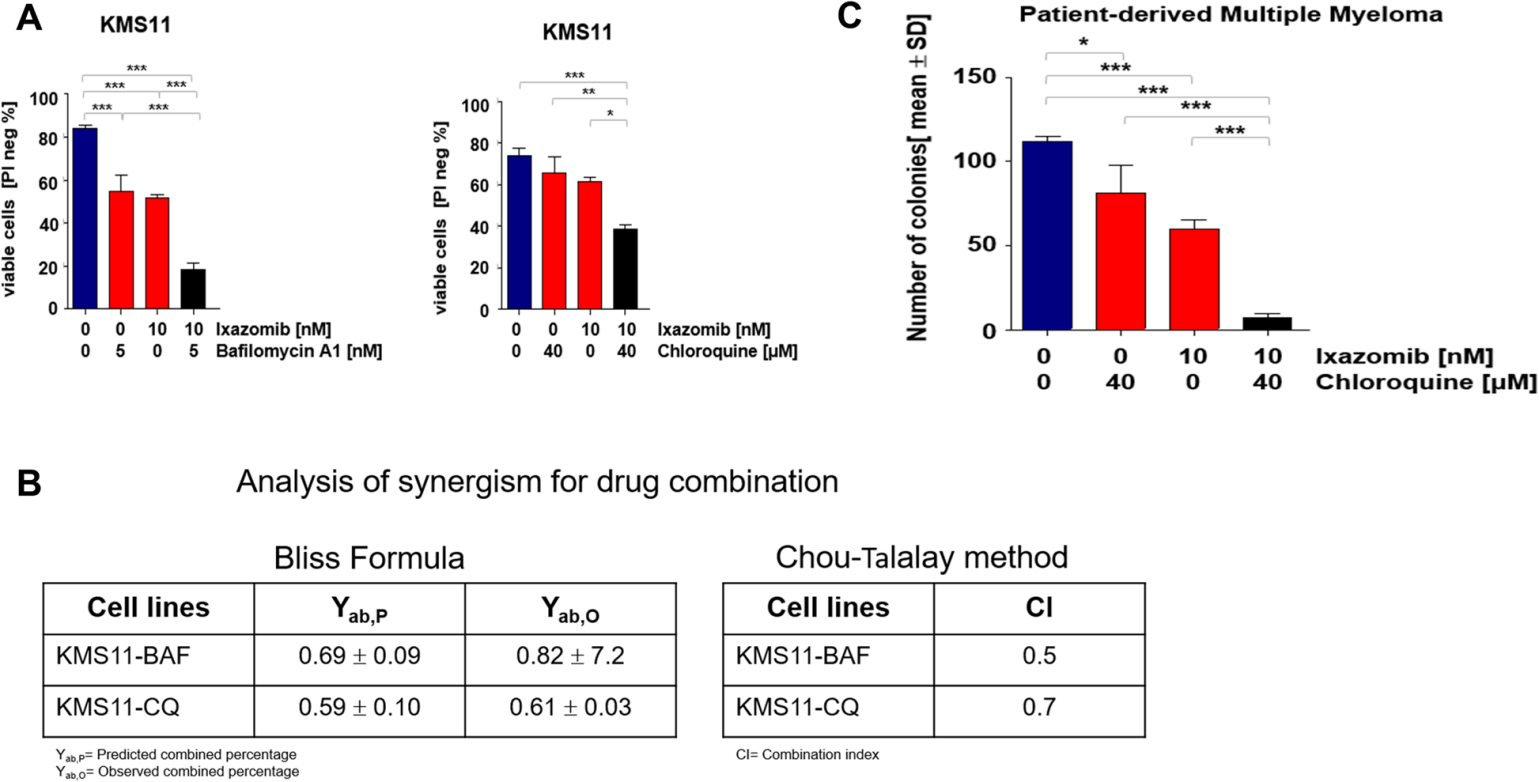
Inhibition of autophagy in combination with ixazomib induces cell toxicity in multiple myeloma cells. KMS11 were treated with 10 nM ixazomib and in combination with autophagy inhibitors bafilomycin A1 (BAF, 5 nM) (*n* = 3) or chloroquine (CQ, 40 μM), (*n* = 2). **A** Cell viability was measured using PI staining after 72 h with BAF and CQ combined treatments respectively. **B** The table represents bliss formula calculation for drug combination analysis. The percentage of inhibitory effect of combined drugs was calculated by bliss formula in comparison with observed inhibitory effect. Based on this formula, we showed that ixazomib in combination with autophagy inhibitors (BAF or CQ) act synergistic. In addition, combination of Index was calculated based on Chou-Talalay. **C** Primary BM MNCs were isolated from multiple myeloma patients at diagnosis (*n* = 7, one representative is shown) and treated with ixazomib 10 nM, chloroquine 40 μM and ixazomib plus chloroquine. After 48 h, 5 × 10.^4^ of all pretreated cells were seeded in CFU culture medium. Colony numbers were counted after 14 days of culture using inverted light microscopy. *P* values were calculated using one-way ANOVA. A *P* value of less than 0.05 was considered statistically significant for all of the analysis (*p < 0.05, **p < 0.01, ***p < 0.001)

Induction of autophagy in response to chemotherapeutic agents is known to protect cancer cells against cell death [29–31]. To determine the antiproliferative effect of ixazomib in combination with the autophagy inhibitors bafilomycin A1 and chloroquine in MM cell lines, we measured cell viability of KMS11 cells upon combination treatment. Autophagy inhibitors combined with ixazomib synergistically reduced viability of KMS11 cells compared to single treatment (Fig. 4A). Using the Chou-Talalay and Bliss formula, interaction efficacy of the two drugs as percentage of cell death mediated by combination treatment was verified (Fig. 4B). We also analyzed the in vitro colony formation of MNCs from bone marrow of patients with multiple myeloma (*n* = 7) after combination treatment. As shown in Fig. 4C, clonogenic growth of MNCs from MM patients was significantly decreased after combination treatment in comparison with monotherapy. However, the number of colonies in two patients after dual therapy showed no significant reduction when compared to single treatment.

### Inhibition of autophagy in combination with ixazomib inhibits cell growth and induces apoptosis

**Fig. 5:**
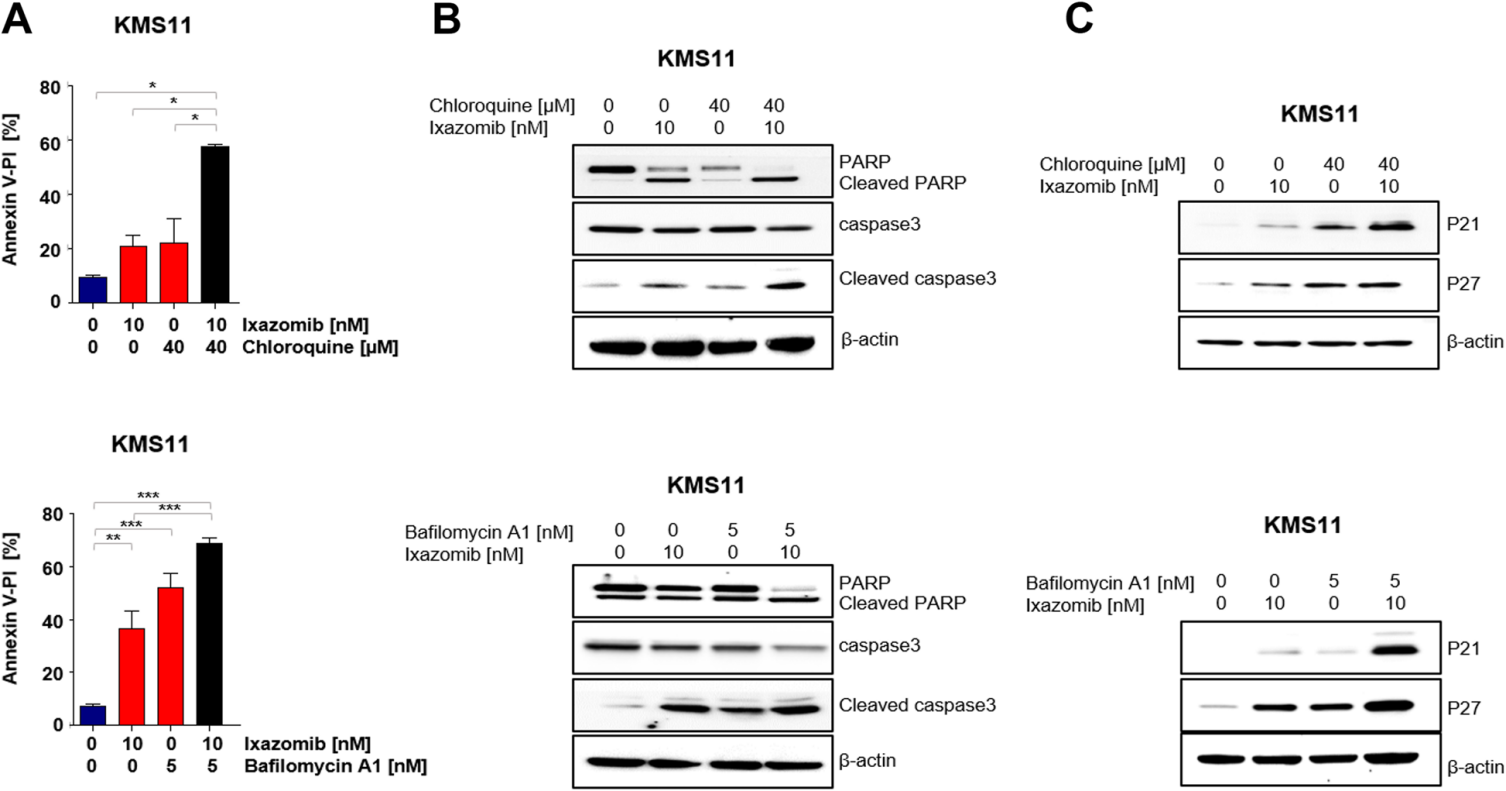
Autophagy inhibitors in combination with ixazomib induce apoptosis and arrest cell cycle at G_1_ phase in multiple myeloma cells. KMS11 cells were treated with ixazomib and autophagy inhibitors as described above. **A** Apoptosis was measured by analysis of apoptosis fraction using annexin-V/PI staining. (BAF, 5 nM) (*n* = 3) or chloroquine (CQ, 40 μM), (*n* = 2). *P* values were calculated by one-way analysis of variance (ANOVA). A *P* value of less than 0.05 was considered statistically significant (*p < 0.05, **p < 0.01, ***p < 0.001). **B** Using western blot protein levels of caspase3, cleaved-caspase3, PARP, cleaved PARP, p21^CIP1^ and p27.^KIP1^ were measured. β-actin was usedas loading control, (*n* = 3)

To determine if bafilomycin A1 (BAF) or chloroquine (CQ) inhibit autophagy, we measured the protein levels of LC3II as marker of autophagy flux. Expression of LC3II was notably increased upon treatment with BAF and CQ (Figure S3), indicating accumulation of LC3II at the autophagosome membrane. Next, we examined whether autophagy inhibitors in combination with ixazomib induce apoptosis in MM cells and determined the apoptotic cell fraction using Annexin-V/PI staining and observed a notable induction of apoptosis compared to single treatment (Fig. 5A). Moreover, we assessed the expression levels of caspase-3 and PARP and their cleaved forms at the protein level. Both autophagy inhibitors in combination with ixazomib increased proteolytic cleavage of caspase 3 and PARP, a signature of activated apoptosis (Fig. 5B). To provide further evidence for induction of apoptosis in MM cells upon combined treatment, we examined its effect on regulation of cell cycle progression. We noticed that expression of G_1_ phase negative regulators p21^CIP1^ and p27^KIP1^ increased after dual treatment, indicating an efficient blockade of cell growth and proliferation in MM (Fig. 5C).

### JNK is activated by the combination of ixazomib and autophagy inhibition and JNK inhibition abrogates the cytotoxic effects of combined treatment

**Fig. 6:**
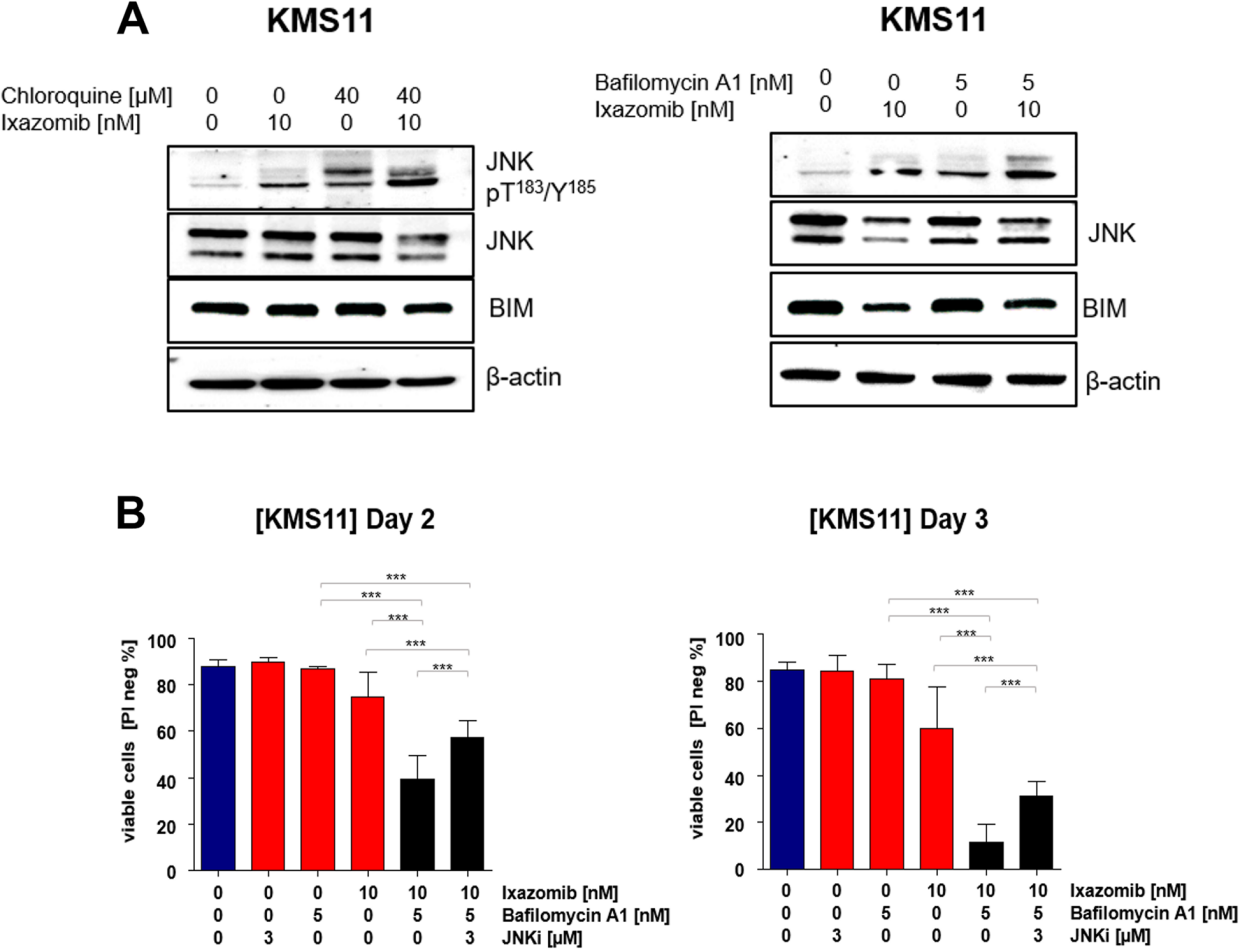
JNK inhibition rescues multiple myeloma cells from cell death mediated by combination treatment. KMS11 were treated with 10 nM ixazomib and in combination with autophagy inhibitors as described above, (*n* = 3). **A** Phosphorylation levels of JNK-T^183^/Y.^185^ and protein levels of JNK and BIM were measured using western blot with β-actin as a loading control. **B** KMS11 cells were treated with 10 nM ixazomib, 5 nM bafilomycin A1, 3 μM JNK inhibitor (JNK-IN-8) and combination treatment (ixa + baf and ixa + baf + JNK). Cell viability of KMS11 cells were measured using PI staining. *P* value were calculated by two-way analysis of variance (ANOVA). A *p* value of less than 0.05 was considered statistically significant (*p < 0.05, **p < 0.01, ***p < 0.001 versus control)

Having shown that ixazomib in combination with autophagy inhibitors induces apoptosis in MM cells, we next speculated that this effect is mediated by the cell stress response as a consequence of dual treatment. JNK kinases have been well characterized as stress activated protein kinases, which can either be induced by extracellular stimuli, protein synthesis inhibitors, cytotoxic drugs or a combination of these mechanisms. Both kinases activate caspase cascades to initiate apoptosis [32–34]. We examined JNK expression in our set of treatments and observed that both autophagy inhibitors BAF and CQ in combination with ixazomib increase phosphorylation levels of JNK in KMS11 cells (Fig. 6A). To analyze the contribution of JNK to apoptosis triggered by autophagy inhibitors and ixazomib, we employed the JNK inhibitor JNK-IN-8 in combination with ixazomib and BAF. Addition of JNK-IN-8 enhanced cell viability compared to ixazomib in combination with only BAF (Fig. 6B), thereby confirming that JNK is an important mediator in cell apoptosis induced by combination treatment in MM cells.

## Discussion

Maintenance of proteostasis plays a pivotal role for the survival and proliferation of MM cells. MM cells are heavily dependent on adaptive pathways to protect them against cell death mediated by stress which is caused by their high metabolic demand for immunoglobulin secretion. Drug discovery has led to the development of various promising candidate drugs for the treatment of MM. Proteasome inhibitors (PIs) with bortezomib, carfilzomib and ixazomib as main representatives of this drug class have tremendously improved the treatment of MM. However, development of resistance and the overall goal of achieving a more profound, ideally complete remission in MM treatment are still considered major challenges. Therefore, developing alternative therapeutic strategies is crucial for MM patients and provided the rationale for the present study.

Recently published data demonstrate the significance of the IRE1α-XBP1 axis of the UPR network in solid tumors and hematologic malignancies [10, 14, 35, 36]. Here, we tested the inhibition of the IRE1α-XBP1 pathway of the UPR in combination with ixazomib as a novel therapeutic approach toward MM. Our data show that IRE1α inhibitors significantly contributed to the cytotoxic effects of ixazomib even when MM cells are protected against the therapeutically efficient drugs by the MSC-mimicked bone marrow microenvironment. Our in vitro results reveal an induction of *XBP1* expression upon ixazomib treatment in MM cells, highlighting the significance of XBP1 as a pro-survival mechanism against the induction of cell death by ixazomib. Apoptosis can be mediated by ER stress through the up-regulation of ER stress responsive genes [27, 28]. We demonstrate that mRNA expression of two pro-apoptotic markers (*PUMA* and *NOXA*) and proteolytic cleavage of PARP strongly increased in combination treatment. Cell cycle analysis revealed that inhibition of XBP1 activation in combination with ixazomib arrests the cell cycle at the G_1_ phase in MM cells. In addition, our finding clearly delineated that blocking XBP1 splicing in combination with ixazomib causes an additive effect on cytotoxicity in MM. Taken together, we demonstrated that ixazomib induces the expression of UPR genes to reduce ER stress. Nevertheless, this response was not limited to the UPR network and other adaptive pathways are activated to compensate for the loss of protein homeostasis upon treatment with ixazomib in MM cells. Autophagy, the other major protein degradation system, also importantly contributed to maintenance of protein homeostasis in our system and we accordingly hypothesized that autophagy might be burdened with an accumulation of proteins for degradation after ixazomib treatment as a compensatory mechanism. This provided a strong rationale for targeting autophagy in combination with ixazomib in MM. We assumed that autophagy is activated to protect MM cells from ixazomib treatment and that, in turn, blocking of autophagy along with ixazomib treatment could maximize cellular toxicity in this context. Recent studies have reported the cytotoxic effects of blockade of the autophagosome-lysosome fusion in cancer cells [37–39]. Here, we showed that both autophagy inhibitors (BAF, CQ) inhibit the late stage of autophagy and lead to increased levels of LC3II and disruption of autophagic flux in MM cells. Our in vitro results verify that the combination of ixazomib and autophagy inhibitors synergistically induces cell death in MM cells. We validated the efficacy of combined treatment in primary case samples from MM patients. In this regard, we revealed that combination treatment induces cell death via apoptosis, as measured by proteolytic cleavage of caspase 3 and its corresponding target PARP. We further demonstrated that our combination therapy is an efficient means to target cell cycle progression. Single agent treatment non-significantly induced cell death through induction of apoptosis, but we were able to show that combination treatment significantly induces cell toxicity in MM cells. The further analyses unveil that activation of JNK is an important molecular mechanism underlying the cytotoxic effects of our combination treatment for MM. However, JNK-mediated signaling has a contentious role across cancer types [40–42]. Studies have implicated that JNK acts in a tumor suppressive manner and is activated in response to cell stress inducers [43, 44]. In line with these findings, our observations hint toward an induction of apoptosis through the activation of stress-responsive protein kinase JNK in MM cells when autophagy inhibitors are combined with ixazomib. Addition of a selective inhibitor of JNK activation together with our dual treatment in turn increased viability of myeloma cells, implicating that activation of JNK in our treatment regimen is a pivotal mechanism for the targeting of MM cells with the combination of proteasome inhibitors and inhibitors of autophagy. A comprehensive investigation of alternative pathways contributing to JNK mediated apoptosis in MM for better insight into the specific role of the JNK signaling pathway and its crosstalk with other crucial molecules in response to targeted treatment of multiple myeloma cells remains to be done. Our present data delineate an innovative strategy against multiple myeloma and clear the way for preclinical and potential clinical investigation of the combination of proteostasis-directed agents in multiple myeloma.

## Supplementary Information

**Additional file 1:** **Figure S1.** Effect of IRE1α inhibitors in combination with ixazomib on cytotoxicity of multiple myeloma cells. **Figure S2.** Ixazomib in combination with A106 arrests cell cycle at G_1_ phase. **Figure S3.** Autophagy inhibitors lead to an accumulation of LC3II. **Table 1.** The list of human primers used for quantitative RT-PCR in this study.

## Data Availability

Extended data are available at Supplementary Material.

## Abbreviations

ATF6: Activating transcription factor 6
BAF: Bafilomycin A1
COX6B: Cytochrome c oxidase subunit 6B1
CQ: Chloroquine
DSMZ: Deutsche Sammlung von Mikroorganismen und Zellkulturen
EIF2AK3: Eukaryotic translation initiation factor 2-alpha kinase 3
IRE1α: Inositol-requiring protein 1 subunit alpha
JNK: C-Jun N-terminal kinase 1
LC3: Microtubule-associated protein light chain 3
MM: Multiple meyloma
NOXA: Alternative name for PMAIP1
PARP: Poly (ADP-ribose) polymerase
PERK: PRKR-Like Endoplasmic Reticulum Kinase
PIs: Proteasome inhibitors
PUMA: P53 up-regulated modulator of apoptosis
SAPK: Stress-activated protein kinase
Ub: Ubiquitin
UPR: Unfolded protein response
UPS: Ubiquitin–proteasome system
XBP1u: X-box-binding protein 1 unspliced
XBP1s: X-box-binding protein 1 spliced
