# Supplementary material for: Targeting autophagy increases the efficacy of proteasome inhibitor treatment in multiple myeloma by induction of apoptosis and activation of JNK
Source: BMC Cancer. 2022 Jul 6;22:735. doi: 10.1186/s12885-022-09775-y (PMC9258169; doi:10.1186/s12885-022-09775-y)

Targeting autophagy increases the efficacy of proteasome inhibitor treatment in multiple myeloma by induction of apoptosis and activation of JNK

Short title: Autophagy and the proteasome in multiple myeloma

Azam Salimi[1,2], Kema Marlen Schroeder[1], Mirle Schemionek-Reinders[1], Margherita Vieri[1], Saskia Maletzke[1], Deniz Gezer[1], Behzad Kharabi Masouleh[1], Iris Appelmann[1+]

[1]Department of Hematology, Oncology, Hemostaseology and Stem Cell Transplantation, Medical Faculty, RWTH Aachen University, Aachen, Germany

[2]Institute of Laboratory Medicine, Universities of Giessen and Marburg Lung Center (UGMLC), Philipps University Marburg, German Center for Lung Research (DZL) Marburg, Germany

[+]Corresponding author:

Dr. Iris Appelmann, MD

Department of Hematology, Oncology, Hemostaseology and Stem Cell Transplantation

RWTH Aachen University Hospital

Pauwelsstrasse 30

52074 Aachen

E-Mail: iappelmann@ukaachen.de

Phone: +49-(0)241-8037358

Fax: +49-(0)241-8082449

Supplementary data

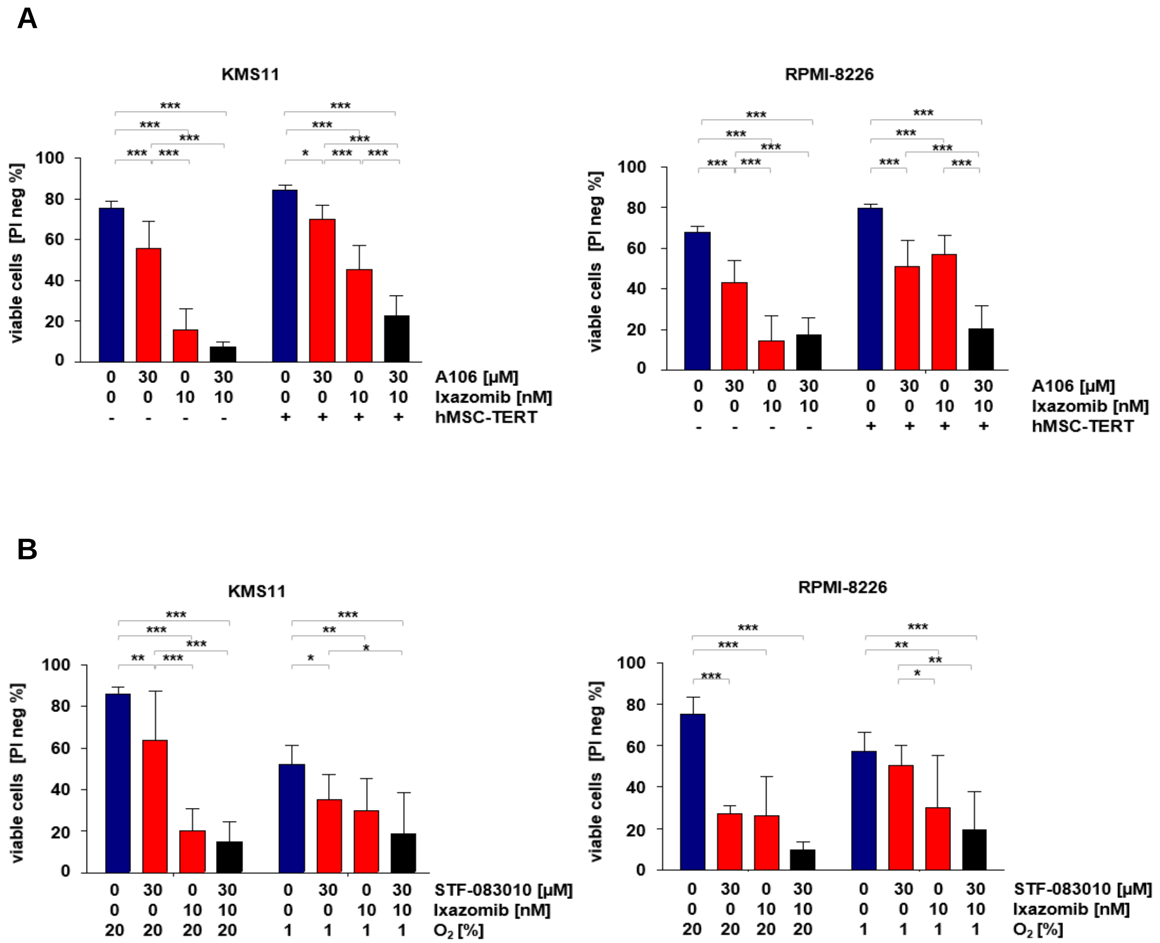

Figure S1. Effect of IRE1α inhibitors in combination with ixazomib on cytotoxicity of multiple myeloma cells. A) KMS11 and RPMI-8226 cells were co-cultured with or without hMSC-TERT following treatment with 10 nM ixazomib as single agent and in combination with 30 µM A106 for five days, n=3. B) KMS11 and RPMI-8226 cells were divided into two groups and were subjected to hypoxic condition (1% O2) versus normoxic condition (20% O2), followed by treatment with 10 nM ixazomib and combined with 30 µM STF-083010. After 5 days cell viability was measured with PI staining, n=3. P values were calculated by two-way analysis of variance (ANOVA). (A) and one-way ANOVA (B). A p value of less than 0.05 was considered statistically significant for all analyses. (*p < 0.05, **p < 0.01, ***p < 0.001 versus control).

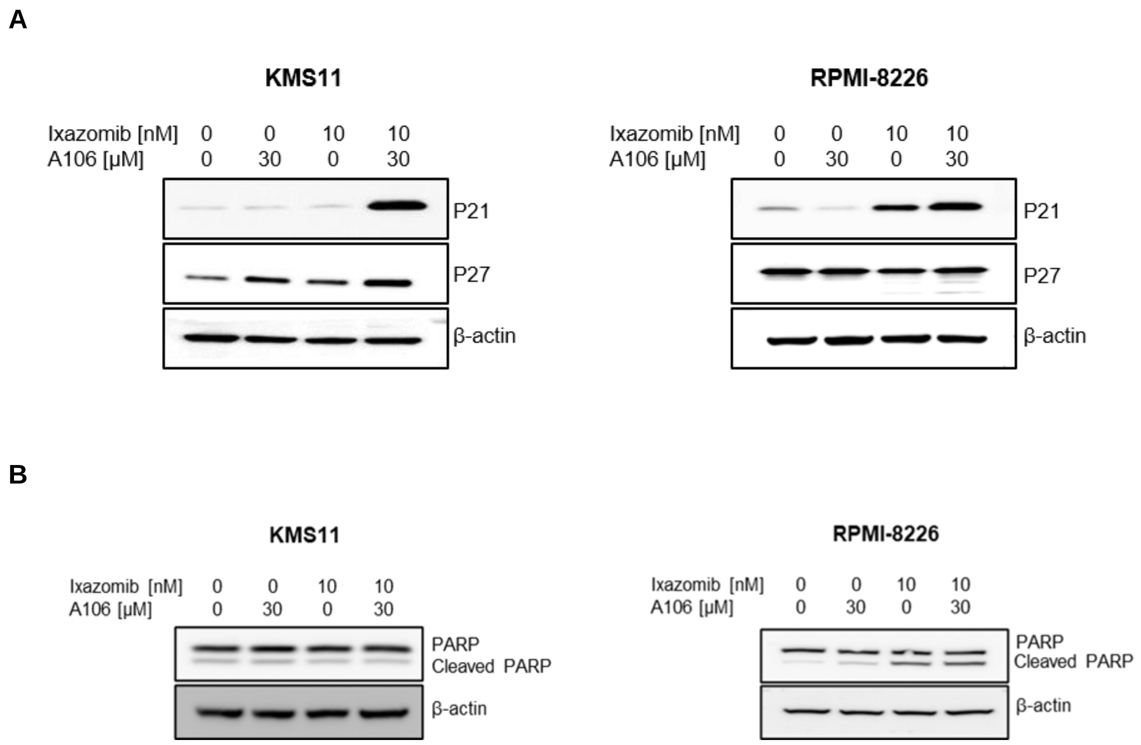

Figure S2. Ixazomib in combination with A106 arrests cell cycle at G_1 phase. KMS11 and RPMI-8226 cells were treated with 10 nM ixazomib as a single agent and in combination with 30 µM A106, n=3. Protein lysates were isolated after 16 hours of treatment. A, B) Protein levels of cell cycle negative regulators $p21^{CIP1}$, $p27^{KIP1}$ and apoptotic markers cleaved-PARP, PARP was measured using western blot. β-actin was used as loading control, n=3.

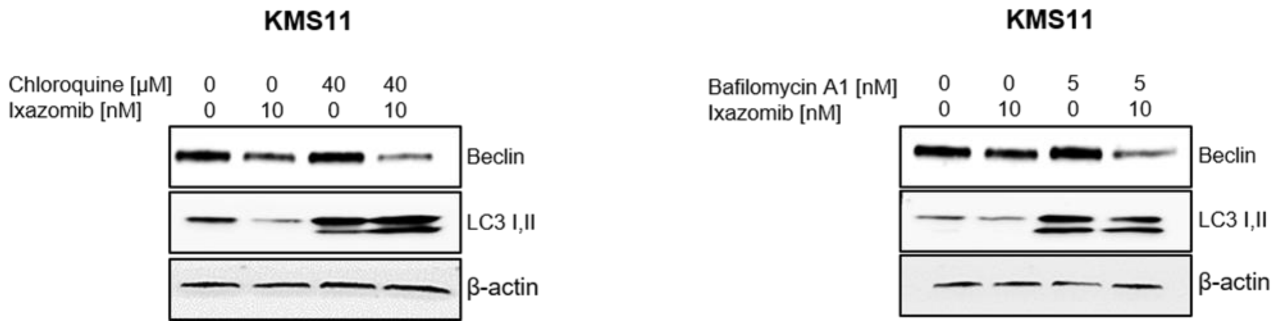

Figure S3. Autophagy inhibitors lead to an accumulation of LC3II. KMS11 cells were treated with 10 nM ixazomib and combined with 5 nM bafilomycin A1 or 40 µM chloroquine and examined for expression levels of LC3I, LC3II and beclin using western blot with β-actin as a loading control, n=3.

Table 1: The list of human primers used for quantitative RT-PCR in this study.

Oligo name	Sequences
COX6B1-F	5'-AACTACAAGACCGCCCCTTT-3'
COX6B1-R	5'-GCTCATCCCAGTCTGTGACC-3'
XBP1s-F	5'-TGACCACATATATACCAAGC-3'
XBP1s-R	5'-GGCTGGATGAAAGCAGATTTGAG-3'
EIF2AK3-F	5'-ATGAGACAGAGTTGCGACCG-3'
EIF2AK3-R	5'-CCCAAATACCTCTGGTTTGC-3'
ATF6-F	5'-TCCTCGGTCAGTGGACTCTTA-3'
ATF6-R	5'-CTTGGGCTGAATTGAAGGTTTTG-3'
PUMA-F	5'-GTCCTCAGCCCTCGCTCT-3'
PUMA-R	5'-TCGTACTGTGCGTTGAGGTC-3'
NOXA-F	5'-CTCTTTCCTCCTCGCCACTT-3'
NOXA-R	5'-GAGTCCCCTCATGCAAGTTT-3'

Additional supplementary data

Figure 2. Ixazomib in combination with STF-083010 arrests cell cycle at G_1 phase.

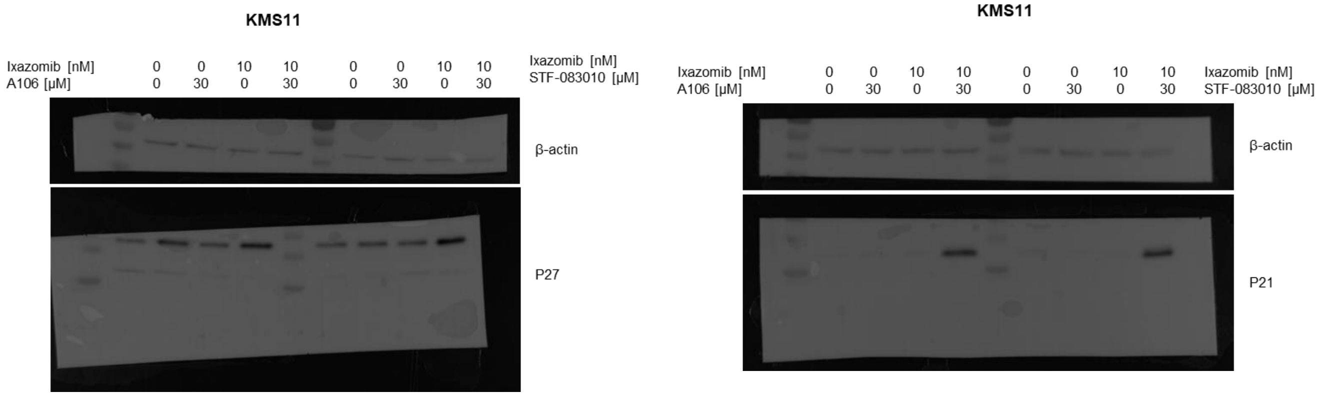

Figure 3. STF-083010 in combination with ixazomib initiates apoptosis in multiple myeloma cell lines.

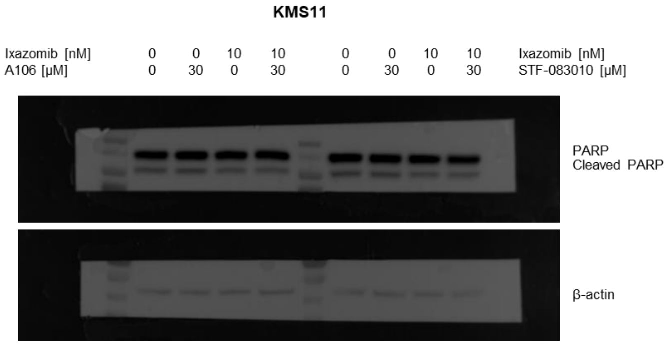

Figure 5. Autophagy inhibitors in combination with ixazomib induce apoptosis and arrest cell cycle at G_1 phase in multiple myeloma cells.

B

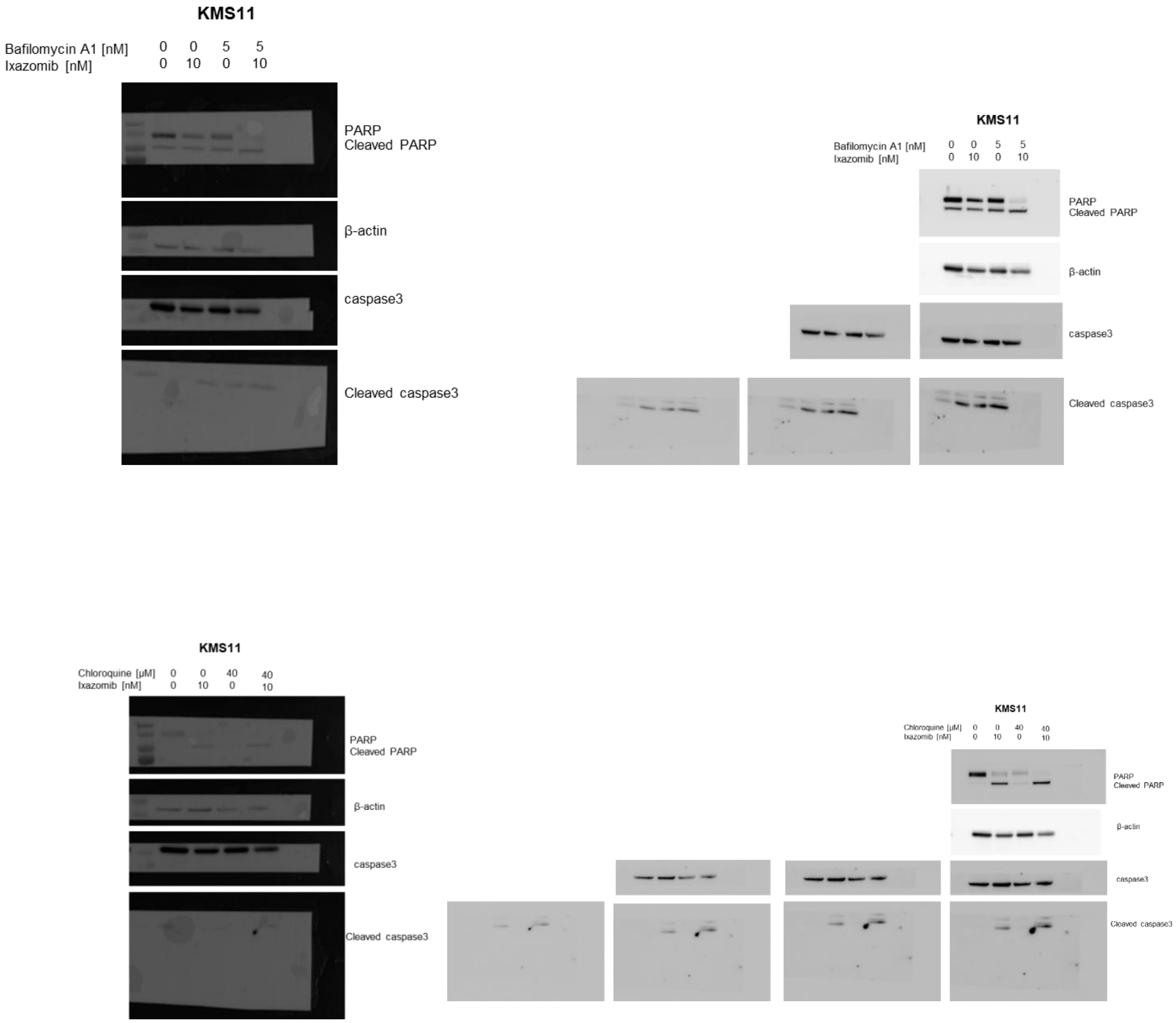

C

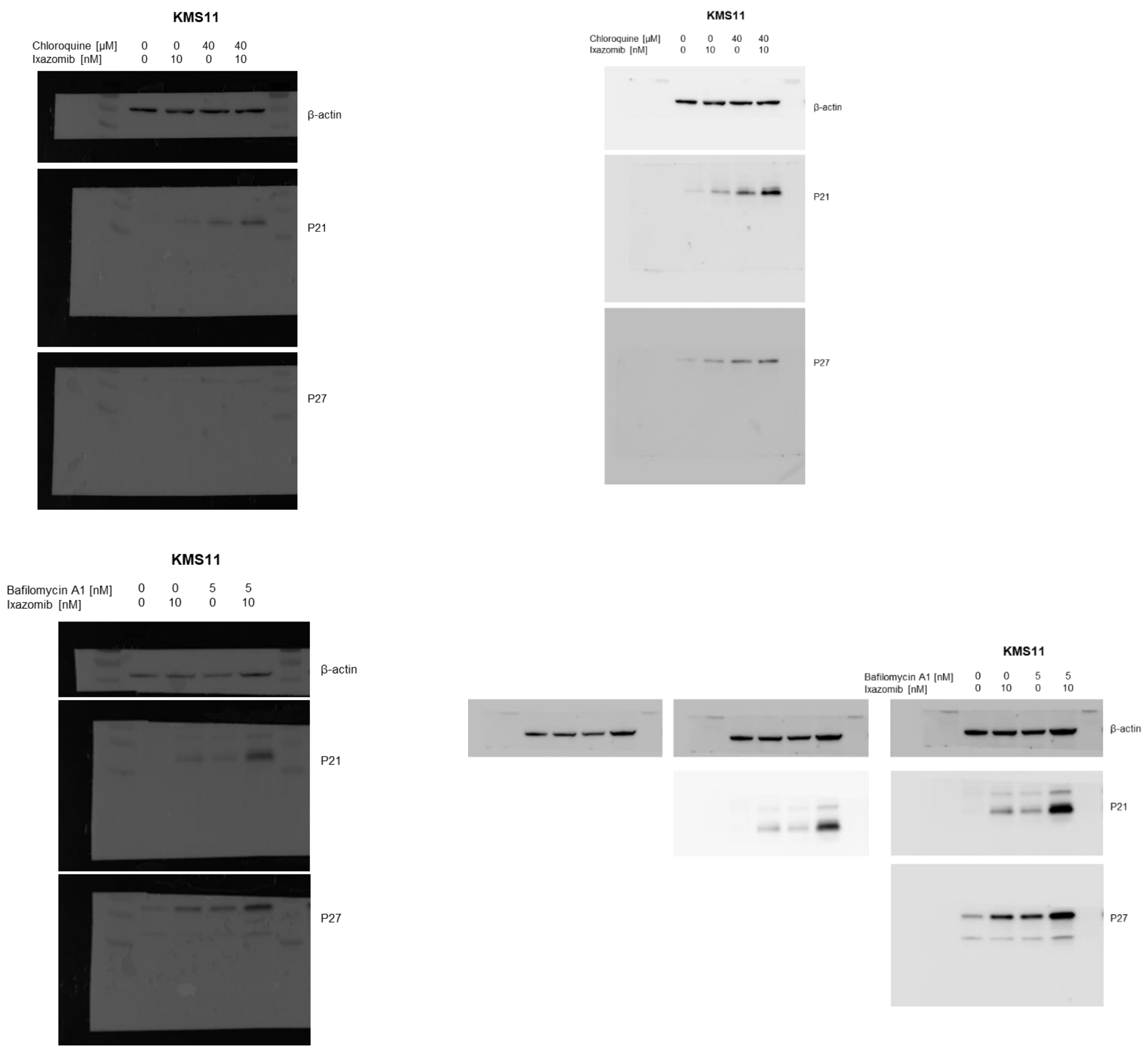
KMS11
Chloroquine [μM] 0 0 40 40
Ixazomib [nM] 0 10 0 10
β-actin
P21
P27
KMS11
Chloroquine [μM] 0 0 40 40
Ixazomib [nM] 0 10 0 10
β-actin
P21
P27
KMS11
Bafilomycin A1 [nM] 0 0 5 5
Ixazomib [nM] 0 10 0 10
β-actin
P21
P27
KMS11
Bafilomycin A1 [nM] 0 0 5 5
Ixazomib [nM] 0 10 0 10
β-actin
P21
P27

Figure 6. JNK inhibition rescues multiple myeloma cells from cell death mediated by combination treatment.

A

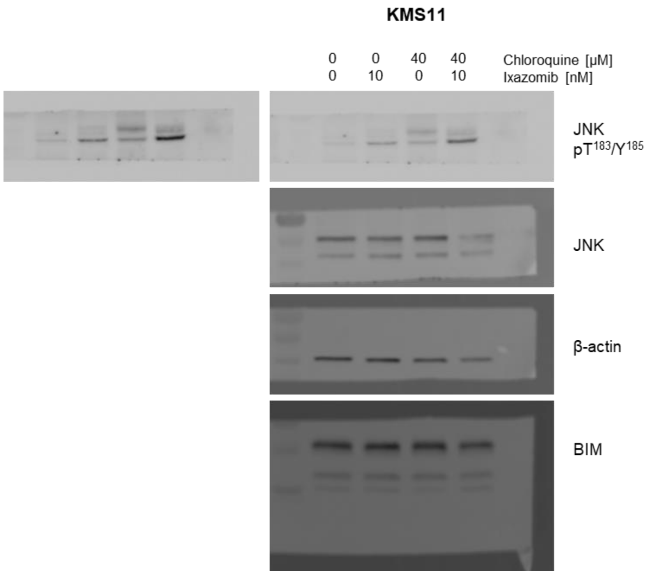

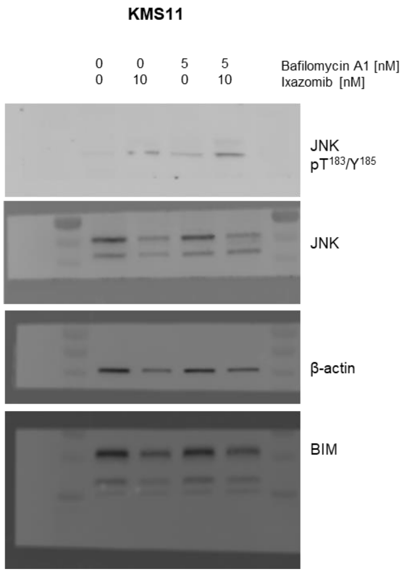

Figure S2. Ixazomib in combination with A106 arrests cell cycle at G_1 phase.

A

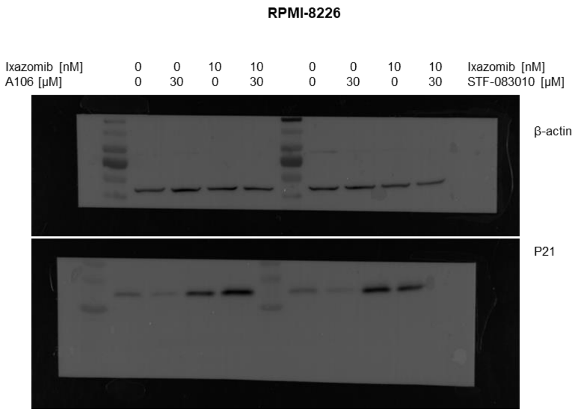

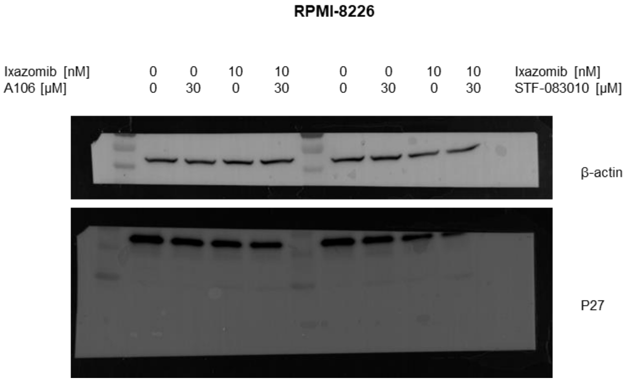

B

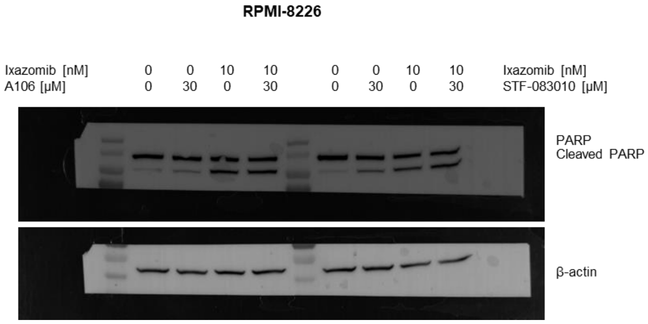

Figure S3. Autophagy inhibitors lead to an accumulation of LC3II.

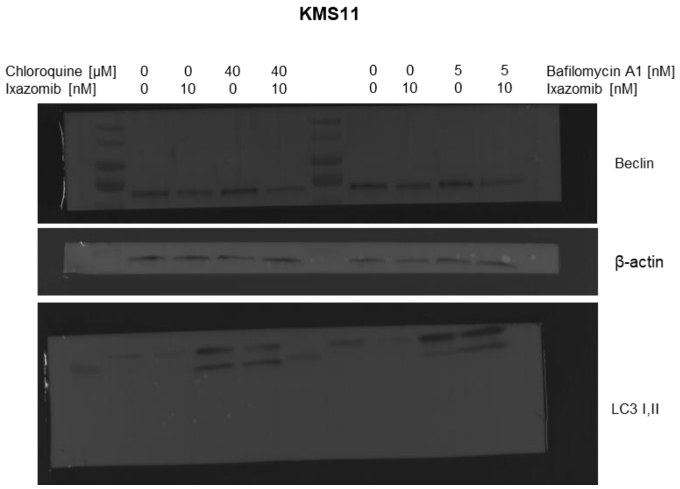

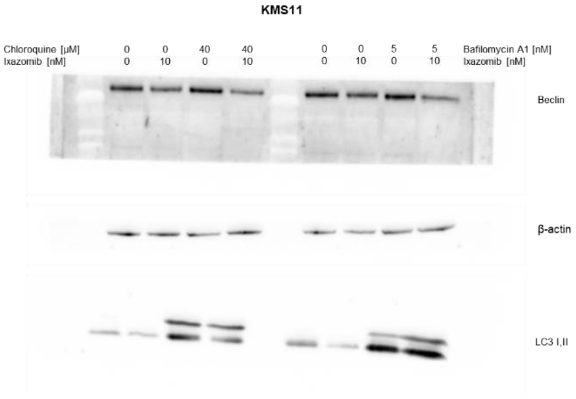

Supplement: Supplementary file 1 — Additional file 1: Figure S1. Effect of IRE1α inhibitors in combination with ixazomib on cytotoxicity of multiple myeloma cells. Figure S2. Ixazomib in combination with A106 arrests cell cycle at G1 phase. Figure S3. Autophagy inhibitors lead to an accumulation of LC3II. Table 1. The list of human primers used for quantitative RT-PCR in this study. [file 12885_2022_9775_MOESM1_ESM.pdf]
